# Epitranscriptomics in myeloid malignancies

**DOI:** 10.1097/BS9.0000000000000124

**Published:** 2022-07-01

**Authors:** Li Han, Jianjun Chen, Rui Su

**Affiliations:** a Department of Systems Biology, Beckman Research Institute of City of Hope, Monrovia, CA; b Department of Pharmacology, School of Pharmacy, China Medical University, Shenyang, China; c City of Hope Comprehensive Cancer Center, City of Hope, Duarte, CA; d Gehr Family Center for Leukemia Research, City of Hope, Duarte, CA

In eukaryotes, gene expression is highly orchestrated not only by genomic promoters and enhancers but also by covalent modifications added to either chromatin or RNAs. Traditionally, “epigenetics” refers to the chemical modifications that govern heritable changes in gene expression independent of the DNA sequence; “epitranscriptomics” indicates the covalent decorations in RNA, which plays a central role in posttranscriptional gene regulation. To date, >170 RNA chemical modifications have been characterized. Most of these modifications were originally identified in highly abundant noncoding RNA species, such as ribosomal RNAs (rRNAs), transfer RNAs (tRNAs), and small nuclear RNA (snRNAs). Recently, the substantial advances in high-throughput sequencing and analytical chemistry have enabled the precise detection and characterization of chemical modifications in messenger RNA (mRNA). Indeed, a considerable number of mRNA decorations have been documented, including N^6^-methyladenosine (m^6^A); N^1^-methyladenosine (m^1^A); N^6^,2′-O-dimethyladenosine (m^6^A_m_); 3-methylcytidine (m^3^C); 5-methylcytidine (m^5^C); 5-hydroxymethylcytidine (hm^5^C); N^4^-acetylcytidine (ac^4^C); Adenosine-to-inosine (A-to-I) editing; pseudouridine (Ψ); N^7^-methylguanosine (m^7^G) and 2′-O-methylated nucleotides (N_m_) (Fig. 1A). The studies from us and other researchers have unveiled that mRNA modifications play important roles in myeloid malignancies.^1–11^ Here, we highlight recent findings focusing on the functions and regulatory mechanisms of mRNA modifications (with an emphasis on m^6^A) and provide our insights to better elucidate epitranscriptomics during leukemogenesis.

**Figure 1.**
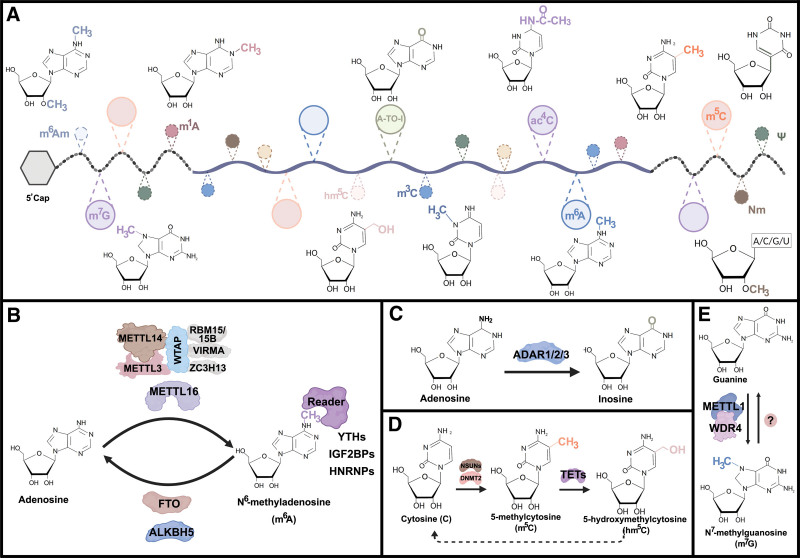
The covalent chemical modifications in mRNA. (A) A schematic view of chemical structures of RNA modifications (m^6^A_m_, m^1^A, A-to-I, ac^4^C, m^5^C, Ψ, m^6^A, hm^5^C, m^3^C, m^7^G and N_m_) in mRNA. (B) The regulation of m^6^A by “writers,” “erasers,” and “readers.” The m^6^A modification is installed by writers, multicomponent m^6^A MTC (composed of METTL3, METTL14, WTAP, RBM15/15B, KIAA1429, and ZC3H13) or METTL16 alone. The two demethylases (eraser), FTO and ALKBH5, remove m^6^A modifications. The m^6^A residue is recognized by the three main classes, including the YTH domain family, IGF2BP family and HNRP family. (C) ADAR enzymes catalyze the A-to-I hydrolytic deamination reaction. (D) The NSUN methyltransferases and DNMT2 catalyze methylation of cytosine-5 and TET family function as dioxygenases catalyzing m^5^C to hm^5^C. (E) The formation of m^7^G is catalyzed by methyltransferases complex, which is composed of METTL1 and WDR4. ADAR = adenosine deaminases acting on RNA.

m^6^A, the most abundant and best-characterized mRNA modification, was initially discovered in 1974.^12^ Owing to the lack of effective and sensitive technologies, recognition of functional significance of m^6^A has been largely absent over the past decades. Until 2011, the identification of fat mass- and obesity-associated protein (FTO) as the first m^6^A demethylase^13^ has profoundly revived the interest in the biological relevance of m^6^A and RNA chemical modifications. Both FTO and AlkB homolog 5 (ALKBH5) can catalyze the demethylation of m^6^A, demonstrating the reversible and dynamic posttranscriptional modification in RNA (Fig. 1B). Methyltransferase-like 3 (METTL3) forms a heterodimer with METTL14 to deposit m^6^A residues in mRNAs. METTL3 acts as the catalytically active methyltransferase with METTL14 serving as an allosteric activator to enhance catalysis.^14^ The large methyltransferase complex (MTC) contains additional subunits, including Wilms tumor 1-associated protein (WTAP), zinc finger CCCH-type contain 13 (ZC33H13), vir like m^6^A methyltransferase associated (VIRMA) and RNA-binding motif protein 15/15B (RBM15/15B) (Fig. 1B). More recently, METTL16 has been identified as a new m^6^A writer, which could exert its enzymatic activity independently and catalyze m^6^A formation on limited mRNAs and noncoding RNAs^15,16^ (Fig. 1B). Characterization of m^6^A “reader” proteins, including YT521-B homology (YTH) domain, insulin-like growth factor 2 mRNA-binding protein (IGF2BP) family, and heterogeneous nuclear ribonucleoprotein (HNRP) family, has provided valuable insights into understanding the underlying mechanism of m^6^A-mediated posttranscriptional gene regulation.

The dysregulation of m^6^A regulators has been reported to be extensively involved in the pathogenesis of myeloid malignancies. We first reported that FTO is highly expressed in acute myeloid leukemia (AML) with t(11q23)/MLL-rearrangements, t(15;17)/PML-RARA, FLT3-ITD and/or NPM1 mutations, and enhances leukemic malignant transformation and leukemogenesis as an m^6^A demethylase.^3^ Subsequently, we showed that R-2-hydroxyglutarate (R-2HG), an oncometabolite of mutant isocitrate dehydrogenase 1/2 (IDH1/2), exerts a broad and intrinsic antileukemic activity through competitively inhibiting the demethylase activity of FTO.^1^ Such identification indicates that FTO might act as a druggable target for leukemia therapy. Moreover, FTO drives and sustains tyrosine kinase inhibitor (TKI) tolerance in leukemia cells by enhancing mRNA stability or translation efficiency of antiapoptotic genes via m^6^A decoration.^17^ Analogous to FTO, the other m^6^A “eraser” ALKBH5 also exerts a tumor-promoting role in leukemia.^4,18^ ALKBH5 is highly expressed in AML, especially in leukemia stem/initiating cells (LSCs/LICs), and maintains LSC/LIC frequency by posttranscriptional regulation of its critical targets, such as TACC3 and AXL.^4,18^ Two independent studies have reported that METTL3 exerts an essential oncogenic role in AML.^6,7^ Mechanistically, METTL3 could deposit m^6^A modification on its mRNA targets, including MYC and BCL2, which, in turn, leads to their translational activation to block myeloid differentiation and promote leukemogenesis.^7^ Another recent study in AML showed that a portion of METTL3 is recruited to the promoter regions of ~80 genes by CAATT enhancers binding protein zeta (CEBPZ) to activate MYC signaling.^6^ METTL14, another essential component of m^6^A MTC, is highly expressed in AML and also plays a critical oncogenic role during leukemogenesis.^5^ Genetic depletion of *METTL14* inhibits cell proliferation, induces myeloid differentiation and cell apoptosis, suppresses self-renewal of LSCs/LICs and delays AML progression In vivo via an m^6^A-deppendent manner.^5^ Several other studies have revealed the oncogenic role of WTAP and RBM15, another 2 components of m^6^A MTC, in leukemia pathogenesis.^19,20^ However, whether such oncogenic roles are attributed to m^6^A decoration remains to be elucidated. Via genome-wide CRISPR-Cas9 screen, METTL16 has been identified as one of the most essential genes for the survival of leukemia cells.^6^ Yet further studies are required to address its role in hematological malignancies. In addition, YTHDF2 is highly expressed in a broad spectrum of human AMLs and required for LSC/LIC self-renewal and AML initiation and propagation via shortening the half-life of m^6^A-modified transcripts.^8^ Another reader protein YTHDC1 is also required for the survival of AML cells.^21,22^ YTHDC1 could undergo liquid–liquid phase separation and form nuclear YTHDC1-m^6^A condensates to protect MYC from the PAXT complex and exosome-associated RNA degradation.^21^ Moreover, YTHDC1 drives leukemogenesis through controlling stability of MCM4.^22^ Taken together with these reported studies on m^6^A regulators, both m^6^A writers and erasers function as oncoproteins in leukemia, despite their opposite role in mRNA methylation. It might be highly possible that different m^6^A writers, especially erasers and readers regulate different groups of target genes, and different m^6^A regulators preferentially bind to distinct regions of the same transcript and result in divergent fates. Actually, IGF2BP preferentially binds to the 3′ UTR region of MYC and increases its stability,^23^ whereas YTHDF2 prefers to bind to the 5′ UTR and middle exons of MYC and leads to its decay.^1^ Thus, it will be very important and interesting to systemically characterize the specific targets of each m^6^A regulator and clarify how a particular m^6^A site in a given transcript fine-tunes its metabolism and determines its fate.

Considering the critical roles of m^6^A modification and its machinery in hematological malignancies, targeting the dysregulated m^6^A regulator(s) may represent an attractive strategy for leukemia treatment. Indeed, several small molecular compounds targeting m^6^A machinery have been discovered. STM2457, a potent and selective inhibitor of METTL3 leads to reduced AML growth and an increase in differentiation and apoptosis.^24^ We have discovered several specific and highly efficient inhibitors targeting FTO to treat leukemia.^25^ More promisingly, m^6^A modification also modulates drug resistance and reprograms immune response.^26^ Therefore, combining such inhibitors targeting m^6^A machinery with chemotherapy and/or immunotherapy may lead to the development of more effective therapies to overcome therapy resistance and cure leukemia.

Aside from m^6^A, other RNA epigenetic marks, such as A-to-I editing, m^5^C and m^7^G, are also implicated in the pathogenesis of hematological malignancies. The adenosine deaminases acting on RNA 1 (ADAR1)-mediated A-to-I editing is correlated with a poor prognosis of chronic myeloid leukemia (CML) patients,^9^ and promotes LSC self-renewal and CML progression (Fig. 1C).^10^ The m^5^C modification in RNA is deposited by m^5^C methyltransferases (RCMTs), including NOL1/NOP2/SUN domain (NSUN) family and DNA methyltransferase homologue (DNMT2), oxidized by TET proteins,^27^ and recognized by Y-box binding protein 1 (YBX1)^28^ (Fig. 1D). NSUN3 and DNMT2 directly interact with hnRNPK to form a functional complex which is important for the survival and drug response of leukemia cells.^11^ Although extensive studies have reported the critical roles of TET protein in blood malignancies, it is totally unknown whether TET-induced demethylation of mRNA m^5^C is involved in leukemogenesis and if so, what the biological function would be. METTL1/WDR4-mediated m^7^G decoration in tRNA potentiates oncogenic transformation and tumorigenesis.^29^ METTL1 also acts as the methyltransferase for internal m^7^G modification in mRNA (Fig. 1E); while it is still underdetermined whether METTL1-induced m^7^G modification in mRNA is involved in leukemogenesis.

Within the past few years, tremendous efforts have been devoted to deciphering the role of RNA modifications in the pathogenesis, leading to rapid expansion of epitranscriptomics. Unlike the well-established methods to investigate m^6^A decoration, studies on other mRNA chemical modifications, such as m^5^C, m^7^G, m^1^A, and m^6^A_m_, are still at their infant stages due to the lack the transcriptome-wide sequencing approaches as well as the characterization of the modification machinery, especially the “eraser” and “reader” proteins. The development of novel tools that can precisely determine the landscape of RNA modifications at single-nucleotide resolution will greatly push the field forward. In addition, further unraveling the fundamental mechanisms of RNA epigenetic modifications and the related machinery may reveal the promising novel therapeutic strategies to treat leukemia and other life-threatening diseases.

## ACKNOWLEDGMENTS

We apologize to colleagues whose work could not be cited due to space limitations.
